# Intravitreal anti-vascular endothelial growth factor monotherapy in age-related macular degeneration with submacular hemorrhage

**DOI:** 10.1038/s41598-023-32874-0

**Published:** 2023-04-07

**Authors:** Maiko Maruyama-Inoue, Yoko Kitajima, Yasuo Yanagi, Tatsuya Inoue, Kazuaki Kadonosono

**Affiliations:** 1 Department of Ophthalmology and Micro-technology, Yokohama City University Medical Center, 4-57 Urafune-Cho, Minami-Ku, Yokohama, Kanagawa 232-0024 Japan; 2 Department of Ophthalmology, Sakae Kyosai Hospital, Yokohama, Kanagawa Japan

**Keywords:** Diseases, Medical research, Risk factors

## Abstract

The purpose of this study was to evaluate the 1-year visual outcomes of patients treated with intravitreal aflibercept (IVA) or brolucizumab (IVBr) for submacular hemorrhage (SMH) secondary to neovascular age-related macular degeneration (AMD). We retrospectively studied 62 treatment-naïve eyes with SMHs exceeding one disc area (DA) secondary to AMD treated with IVA or IVBr. All patients received three monthly intravitreal injections in the loading phase followed by as-needed injections or fixed dosing. If a vitreous hemorrhage (VH) developed during the follow-up period, injections were discontinued and vitrectomy was performed. We evaluated the changes in the best-corrected visual acuity (BCVA) and factors that affected the BCVA improvement and VH development. A VH during treatment developed in five eyes (8.1%) (VH + group), and the mean BCVA worsened from 0.45 to 0.92. The BCVA improved significantly (*P* = 0.040) in the remaining 57 eyes (VH − group) from 0.42 to 0.36. The development of VHs was associated with significantly (*P* < 0.001) less VA improvement. Furthermore, large DAs and younger age at baseline were associated significantly (*P* = 0.010 and 0.046, respectively) with the development of VHs. Both IVA and IVBr appeared to improve functional outcomes in patients with SMH secondary to AMD when VHs did not develop. However, a VH developed in 8.1% of eyes after treatment. Although anti-vascular endothelial growth factor treatments were well-tolerated, for cases with large SMH at baseline, it should be considered that VH may occur during the monotherapy treatment process using IVA or IVBr, and that achieving good visual outcomes may be difficult in some cases.

## Introduction

A large submacular hemorrhage (SMH) is an uncommon manifestation in patients with neovascular age-relater macular degeneration (AMD)^1^. However, knowledge of the natural history of SMH is poor^2,3^. Iron or hemosiderin toxicity to the retina^4,5^, the diffusion barrier between the retina and the retinal pigment epithelium (RPE), and mechanical damage caused by fibrin clots^6^ seem to be associated with mechanisms that cause visual impairment. Therefore, it is important to remove SMHs as soon as possible.

Pneumatic displacement^7^, vitrectomy with gas tamponade^8^, use of tissue plasminogen activator (tPA)^9^, intravitreal anti-vascular endothelial growth factor (VEGF) agents^10^, and combinations of these treatment have been reported in patients with SMH. Although evidence of the safety of these treatments is available, invasive treatments such as pneumatic displacement or vitrectomy exert a greater physical burden on patients. However, anti-VEGF treatment such as ranibizumab (Lucentis, Genentech, Inc., South San Francisco, CA) and aflibercept (Eylea, Bayer Health Care, Berlin, Germany) have had beneficial effects and are less invasive in patients with SMHs secondary to AMD^10,11^. Recently, brolucizumab (Beovue, Novartis Pharmaceuticals, Basel, Switzerland) was approved as a new anti-VEGF agent for AMD. Intravitreal brolucizumab (IVBr) provides better control of intraretinal, subretinal, and sub-RPE fluids than intravitreal aflibercept (IVA)^12^. Therefore, the effectiveness of IVBr for SMHs due to AMD in a real-world clinical setting is anticipated.

The purpose of this study was to evaluate the 1-year visual outcomes of patients treated with IVA or IVBr for SMHs secondary to neovascular AMD and investigate the factors that affected the visual outcomes.

## Results

Sixty-two eyes of 62 patients (45 men, 17 women; mean age, 75.0 ± 9.8 years; range, 48–95 years; 25 right eyes and 37 left eyes) had a SMH secondary to neovascular AMD and were assessed at the 12-months follow-up examination. The baseline patient characteristics and clinical data are shown in Table 1.

**Table 1:** Clinical characteristics of the patients with SMH secondary to neovascular AMD.

Number of patients	62
Number of eyes (right/left)	62 (25/37)
Age, mean ± SD, year	75.0 ± 9.8
Sex (male/female) (%)	45(73)/17 (27)
Anti-VEGF, aflibercept/brolucizumab (%)	46(74)/16(26)
The subtype of AMD (PCV/non-PCV) (%)	39(63)/23 (37)
Anticoagulant medication (+ /−) (%)	6(10)/56(90)
Duration of symptoms (days)	27.4 ± 20.9 (range, 2–90)
Mean size of submacular hemorrhage (DAs)	3.0 ± 2.0 (range, 1–11)
Vitreous hemorrhage during treatment, n (%)	5(8.1)
Mean baseline logMAR BCVA	0.43 ± 0.36
Mean number of anti-VEGF injections	6.0 ± 2.3 (range, 1–12)
Mean central foveal thickness (µm)	567 ± 245
Mean thickness of SMH at the fovea (µm)	214 ± 167
Mean thickness of hemorrhagic PED at the fovea (µm)	201 ± 253

*SMH* submacular hemorrhage, *AMD* age-related macular degeneration, *SD* standard deviation, *VEGF* vascular endothelial growth factor, *PCV* polypoidal choroidal vasculopathy, *DAs* disc areas, *BCVA* best-corrected visual acuity, *PED* pigment epithelial detachment.

### Development of VH

**Figure 1:**
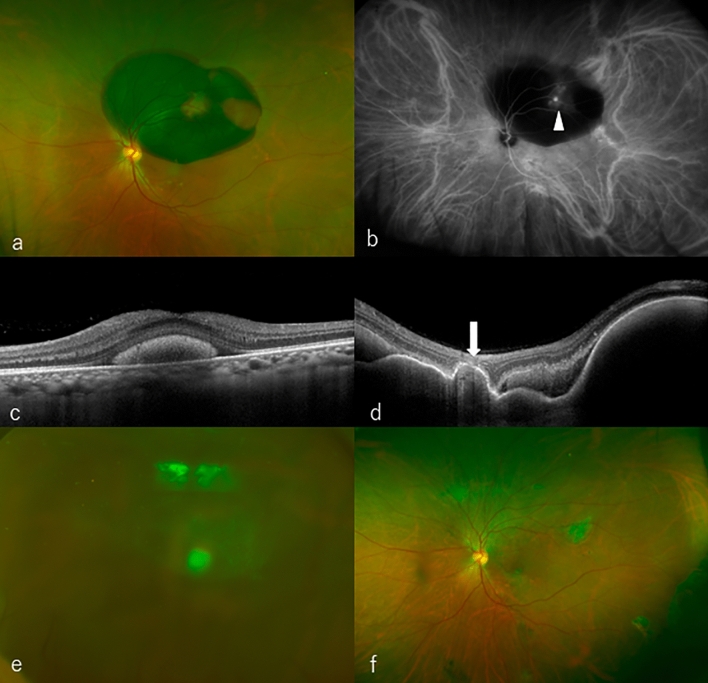
(**a**) A 63-years-old man presented with visual loss in his left eye (BCVA 20/20). Funduscopic examination shows a SMH including the macula. (**b**) Indocyanine green angiography (ICGA) shows blockage due to the hemorrhage and the hyperfluorescent lesions (arrowhead). (**c**) A baseline OCT image shows the SMH. (**d**) SD-OCT corresponding to the hyperfluorescent lesions in ICGA shows the protrusion of polyps (arrow) with the SMH and a hemorrhagic PED. He was diagnosed with PCV and started IVBr treatment. (**e**) One month after the initial treatment, VH developed and his BCVA decreased to 20/300. Surgical treatment was performed. (**f**) Fundus photography at 12 months shows that the SMH improved although his BCVA decreases to 20/200.

A vitreous hemorrhage (VH) developed in five eyes (8.1%) after treatments (VH + group). Three eyes had VH at 1 month after an initial treatment, and two eyes had VH at 7 months after the initial treatment. No VH developed in the remaining 57 eyes (VH-group) during the follow-up examinations. Table 2 shows the comparison of the clinical characteristics between the VH + and VH − groups. There were no significant differences in sex, type of anti-VEGF agents, AMD subtype, proportion of patients taking anticoagulants, baseline BCVA, mean central foveal thickness (CFT), mean thickness of the SMHs, and mean thickness of the hemorrhagic pigment epithelial detachment (PED)s between the two groups (*P* > 0.05 for all comparisons). However, the patients in the VH + group were younger, and the disc area (DA)s were larger (*P* < 0.05 for both comparisons). Figure 1 shows the case that underwent vitrectomy due to VH occurrence during the treatment process.

**Table 2:** Comparison between VH + group and VH − group.

	VH + group (n = 5)	VH − group (n = 57)	*P*-value*
Number of patients	5	57	
Sex (male/female)	4/1	41/16	1.000
Age, mean ± SD, year (range)	62.8 ± 11.2 (range, 48–79)	76.0 ± 9.1 (range, 53–95)	0.022
Anti-VEGF, aflibercept/brolucizumab	3/2	43/14	0.597
The subtype of AMD (PCV/non-PCV)	4/1	35/22	0.643
Anticoagulant medication (+ /−)	0/5	6/51	1.000
Mean size of submacular hemorrhage (DAs)	7.2 ± 3.6 (range, 2.6–11)	2.7 ± 1.5 (range, 1–8.5)	0.014
Mean baseline logMAR BCVA	0.45 ± 0.45	0.42 ± 0.36	0.846
Mean central foveal thickness (µm)	573 ± 171	567 ± 252	0.614
Mean thickness of SMH at the fovea(µm)	299 ± 194	207 ± 165	0.187
Mean thickness of hemorrhagic PED at the fovea (µm)	111 ± 155	209 ± 259	0.337

*SMH* submacular hemorrhage, *AMD* age-related macular degeneration, *SD* standard deviation, *VEGF* vascular endothelial growth factor, *PCV* polypoidal choroidal vasculopathy, *DAs* disc areas, *BCVA* best-corrected visual acuity, *PED* pigment epithelial detachment.

*P-value calculated using Man-Whitney U test and the Fisher’s exact test.

### Changes in the BCVA

**Figure 2:**
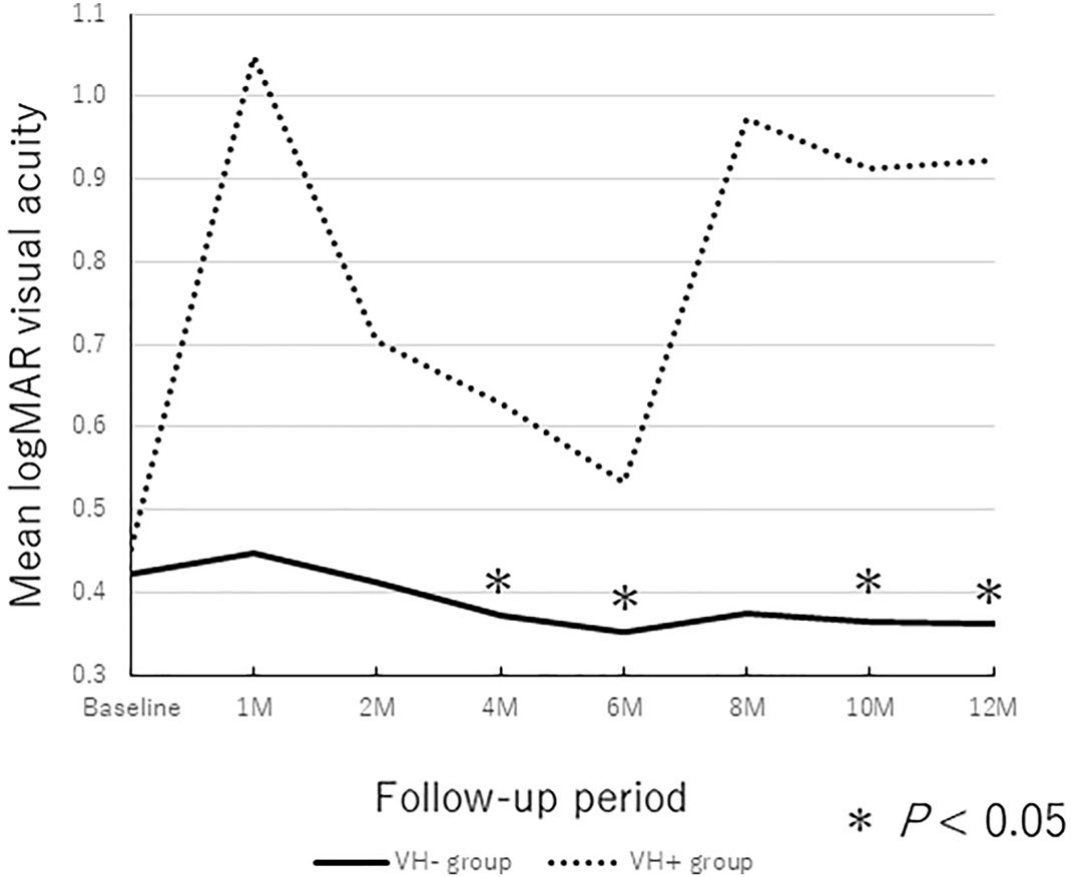
Changes in the BCVA during the 12-months follow-up period. The mean BCVA at 4, 6, 10, and 12 months improved significantly compared with those at baseline in VH − group (*P < 0.05).

The baseline mean logarithm of the minimum angle of resolution (logMAR) VAs were 0.42 ± 0.36 in the VH − group and 0.45 ± 0.45 in the VH + group. The mean logMAR BCVAs at 6 and 12 months in both groups after the initial treatment, respectively, were 0.35 ± 0.45 and 0.39 ± 0.48 in the VH − group and 0.53 ± 0.39 and 0.92 ± 0.51 in VH + group. In the VH − group, the post-injection BCVA improved significantly compared with preoperatively (*P* = 0.018 and* P* = 0.040 at 6 and 12 months, respectively) (Fig. 2). However, in the VH + group, the post-injection BCVAs at 6 and 12 months did not change significantly compared with baseline (*P* = 1.000, and *P* = 0.138 at 6 and 12 months, respectively) and the VH + group showed a trend toward visual worsening during the follow-up period.

### Factors affecting the BCVA improvement at 12 months

Multiple linear regression (Table 3) indicated that VH development was the only factor that was associated significantly (*P* < 0.001) with a worse BCVA at 12 months. No significant correlations were observed between age, sex, type of anti-VEGF, use or no use of anticoagulants, AMD subtype, symptom duration, DAs, baseline BCVA, CFT, thickness of the SMHs at the fovea, thickness of hemorrhagic PEDs at the fovea, and the changes in the BCVA (*P* > 0.05 for all comparisons).

**Table 3:** Stepwise multiple regression analysis of the improvement of the BCVA.

Independent variables	Dependent variable		
	Improvement of the visual acuity*		
	Partial regression coefficient	Standard error	*p* value
Age	0.0058	0.0042	0.1709
Development of VH	0.6074	0.1491	< 0.001

Excluded variables: sex, type of anti-VEGF agents, taking anticoaglants, subtype of AMD, duration of symptoms, DAs, baseline BCVA, CFT, thickness of SMH, and thickness of PED.

*BCVA* best-corrected visual acuity, *VH* vitreous hemorrhage, *VEGF* vascular endothelial growth factor, *AMD* age-related macualr degeneration, *DAs* disc areas, *CFT* central foveal thickness, *SMH* submacular hemorrhage, *PED* pigment epithelial detachment.

*Improvement of the visual acuity = the difference between the baseline visual acuity and postinjection BCVA at 12 months.

### Factors affecting VH development

Stepwise logistic analyses (Table 4) indicated that larger DAs (odds ratio [OR] 1.972; 95% confidence interval [CI] 1.180–3.297; *P* = 0.010), and younger age (OR, 0.839; 95% CI 0.706–0.997; *P* = 0.046) were associated significantly with VH development. However, no significant correlations were seen between sex, type of anti-VEGF agents, use or no use of anticoagulants, AMD subtype, symptom duration, CFT, thickness of the SMHs at the fovea, thickness of the hemorrhagic PEDs at the fovea, and the presence of a VH (*P* > 0.05 for all comparisons).

**Table 4:** Logistic analyses which investigate factors influencing vitreous hemorrhage.

Variables	Multivariate analysis		*P*-value
	Odds ratio	95% CI	
Age	0.839	0.706–0.997	0.046
DAs	1.972	1.180–3.297	0.010

*CI* confidential interval, *DAs* disc areas.

Excluded variables: sex, anti-VEGF, + /− of taking anticoaglant, subtype of AMD, duration of symptomes, CFT, the thickness of SMH, and the thickness of hemorrhagic PED.

**Figure 3:**
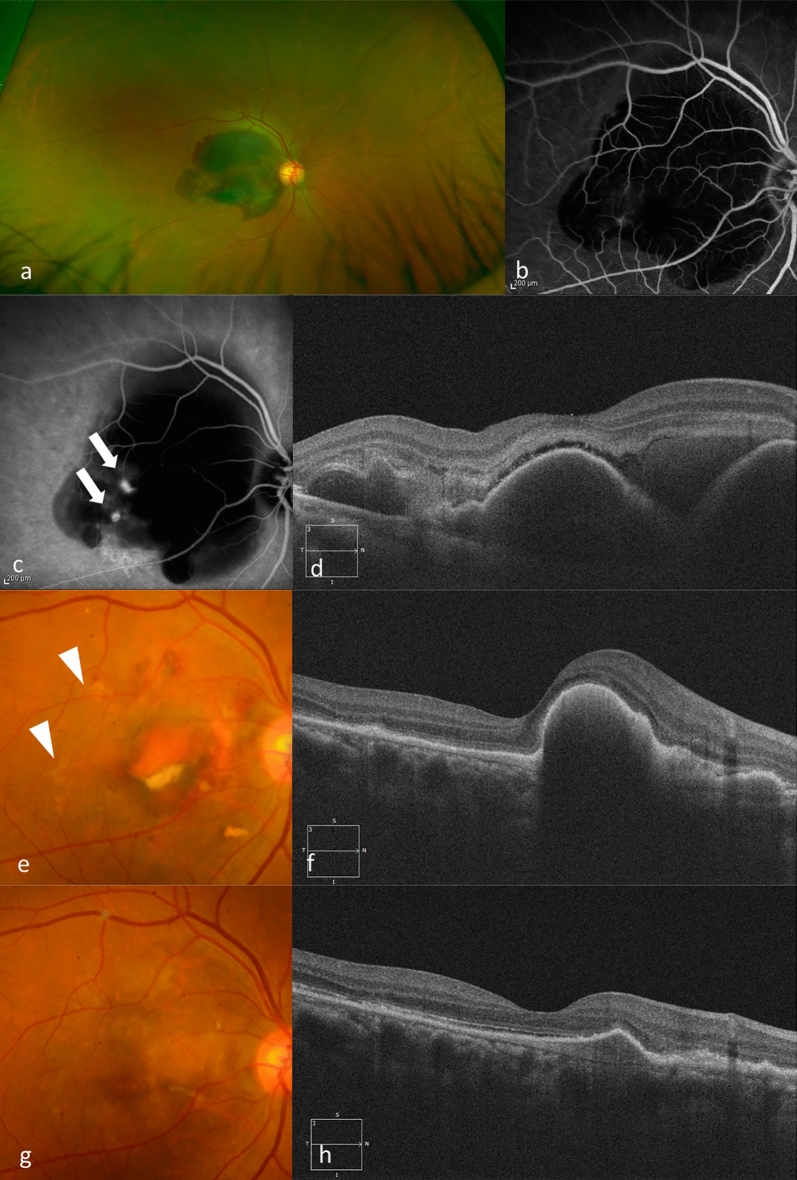
(**a**) A 60-years-old woman presented with sudden visual loss in her right eye (BCVA 20/30). Funduscopic examination in that eye shows a SMH in the macula. (**b**) Fluorescein angiography shows blockage due to the hemorrhage. (**c**) Indocyanine green angiography shows a polypoidal lesion and an abnormal vascular network (arrows). (**d**) A baseline OCT image shows a SMH and hemorrhagic PED. She was diagnosed PCV and received IVA treatment monthly during the loading phase. (**e**) Fundus photography at 3 months shows that the SMH resolved. Hyperpigmented spots are noted (arrowheads). (**f**) OCT at 3 months still shows a hemorrhagic PED. (**g**) Fundus photography at 1 year shows no SMH. Her BCVA improved to 20/20. (**h**) SD-OCT at 1 year shows improvement of the hemorrhagic PED.

**Figure 4:**
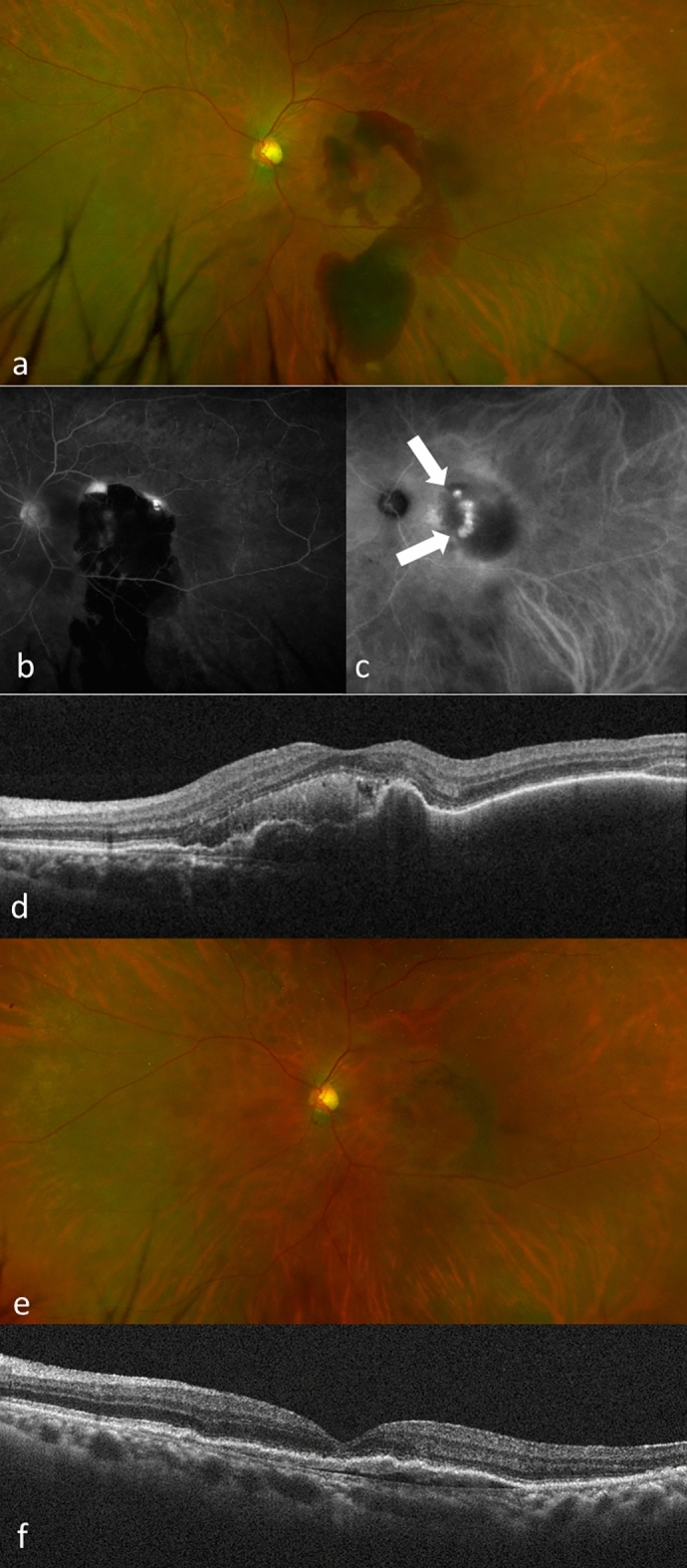
(**a**) A 67-years-old man presented with visual loss in his left eye (BCVA 20/100). Examination shows a SMH in the macula extending outside the vascular arcade. (**b**) Fluorescein angiography shows blockage due to the hemorrhage and hyperfluorescent area. (**c**) Indocyanine green angiography shows a polypoidal lesion (arrows). (**d**) A baseline OCT image shows a SMH at the fovea and hemorrhagic PED. He was diagnosed with PCV. He received IVBr treatment during the loading phase and received IVBr every 2 months during the maintenance phase. (**e**) Fundus photography at 1 year shows improvement of the SMH. (**f**) OCT at 1 year shows resolution of the SMH and reduced PED. The BCVA improved slightly to 20/63.

Figures 3 and 4 show the results for eyes with large SMHs treated with aflibercept and brolucizumab, respectively.

### Sub-analysis of PCV patients

**Figure 5:**
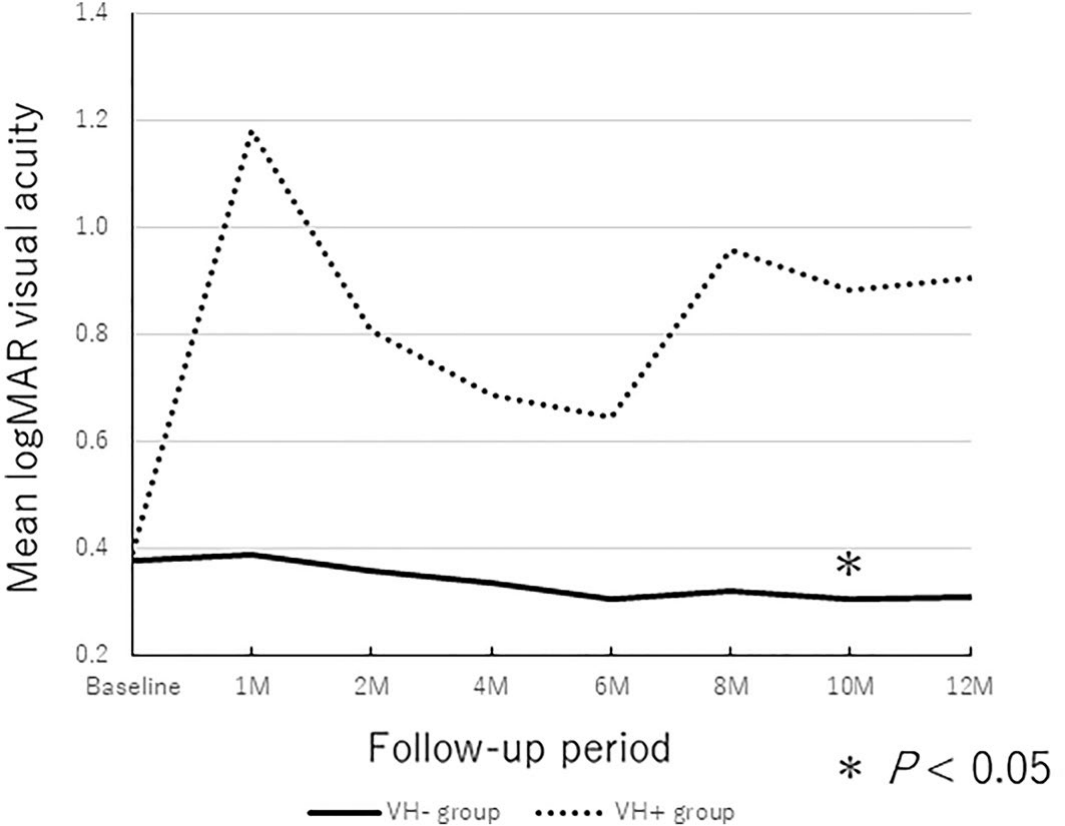
Changes in the BCVA during the 12-months follow-up period in patients with PCV. Only the mean BCVA at 10 months improved significantly compared with those at baseline in VH − group (*P < 0.05).

Thirty-nine eyes (62.9%) had polypoidal choroidal vasculopathy (PCV). Among the 39 eyes, 29 eyes (74.4%) were men and 10 eyes (25.6%) were women. A VH developed in 4 eyes (10.3%). The baseline mean logMAR VAs were 0.38 ± 0.36 in the VH − group and 0.39 ± 0.50 in the VH + group. The mean logMAR BCVAs at 6 and 12 months in both groups after the initial treatment, respectively, were 0.31 ± 0.44 and 0.31 ± 0.43 in the VH − group and 0.64 ± 0.35 and 0.91 ± 0.59 in VH + group. In the VH − group, the post-injection BCVA tended to improve although a significant difference was seen only in the 10 months (*P* = 0.036 at 10 months) (Fig. 5). On the other hand, in the VH + group, the post-injection BCVAs did not change significantly compared with baseline throughout 12-months period. Multiple linear regression indicated that VH development was the only factor that was associated significantly (*P* < 0.001) with a worse BCVA at 12 months. No significant correlations were observed between age, sex, type of anti-VEGF, use or no use of anticoagulants, symptom duration, DAs, baseline BCVA, CFT, thickness of the SMHs at the fovea, thickness of hemorrhagic PEDs at the fovea, and baseline BCVA and the changes in the BCVA (*P* > 0.05 for all comparisons). That is, sub-analysis of PCV patients was similar to the results of all patients.

### Occurrence of hyperpigmented spots

Among 62 eyes, 26 eyes (41.9%) showed hyper pigmented spots after the induction therapy. Stepwise logistic analyses indicated that larger DAs (odds ratio [OR] 1.708; 95% confidence interval [CI] 0.103–0.968; *P* = 0.015), and VH development (OR 50.014; 95% CI 0.090–7.735; *P* = 0.045) were associated significantly with having hyperpigmented spots. No significant correlations were seen between age, sex, type of anti-VEGF agents, use or no use of anticoagulants, AMD subtype, symptom duration, DAs, baseline BCVA, CFT, thickness of the SMHs at the fovea, thickness of the hemorrhagic PEDs at the fovea, and the presence of hyperpigmented spots (*P* > 0.05 for all comparisons).

## Discussion

The results showed that at the 1-year follow-up examinations, IVA and IVBr improved vision in treatment-naïve patients with SMHs secondary to AMD unless VHs developed. Also, sub-analysis study exhibited that patients with PCV also showed the same result. However, our retrospective investigation showed that VHs developed after treatments at an estimated incidence rate of 8.1%, which resulted in worse visual outcomes. Physicians should especially consider that larger DAs and younger age are risk factors for the development of VHs. The current report is the first to describe the functional outcomes of IVBr in patients with treatment-naïve AMD with a SMH. No significant difference was seen between IVA and IVBr in the visual outcomes after treatment.

Anti-VEGF treatments such as ranibizumab and aflibercept, which are used worldwide, are effective in patients with neovascular AMD^13,14^. However, for example, the ANCHOR/MARINA studies, which were randomized controlled trials of anti-VEGF therapies for AMD, excluded patients with SMHs occupying more than 50% of the lesion area^13,15^. In addition, no randomized controlled trial has evaluated anti-VEGF monotherapy for SMHs associated with AMD. However, using anti-VEGF treatments to treat SMHs secondary to AMD seems reasonable to suppress the activity of neovascular lesions and help resorb the hemorrhage. Recently, the efficacy of anti-VEGF monotherapies such as bevacizumab (Avastin, Genentech Inc.), ranibizumab, and aflibercept, in patients with SMHs associated with AMD has been reported^11,16^. In the current study, patients who completed the follow-up period without development of VHs (91.9%) had significantly improved BCVA with aflibercept and brolucizumab, which was consistent with previous studies.

However, a VH developed in 8.1% of patients after treatment and these patients had worse BCVA at 12 months. Although the BCVA in VH + group tended to improve from 1 to 6 months, it deteriorated again because two eyes developed VH at 7 months after the initial treatment. Kim et al.^16^ reported that bevacizumab and ranibizumab resulted in improved logMAR BCVA from 1.14 to 0.82 in patients with SMHs secondary to AMD. However, in that study a VH developed in 20.4% of patients. Kim et al.^17^ enrolled 91 patients to analyze the efficacy of ranibizumab, but they excluded 11 patients in whom a VH developed beforehand. Kim et al.^11^ also described that one of 30 eyes (3.3%) treated with aflibercept develop a VH. Although a direct comparison between the current study and previous studies is difficult, physicians should be aware that a VH can develop in some patients with SMHs. In the current study, there was no difference in the proportion of development of VHs between aflibercept and brolucizumab; however, larger prospective studies are needed to investigate the differences between aflibercept and brolucizumab.

In the current study, larger DAs at baseline and younger age were associated significantly with the development of a VH by stepwise logistic analyses. There were also significant differences in the mean size of SMHs at baseline and age between VH + group and VH − group. However, the odds ratio of age was relatively low and the effect of age on VH development may be less than the effect of DAs. Kim et al.^16^ reported that larger DAs, greater CFT, and the presence of a PED at the fovea were associated with a higher risk of developing a VH. Shin et al.^18^ reported that taking anticoagulant medications, larger SMHs, and PCVs were risk factors for development of a VH. Considering their studies, large SMHs seem to be associated strongly with the development of VHs. The current study also showed that the development of VHs resulted in worse VA at 1 year. Therefore, patients with large hemorrhages at baseline should be paid attention to a high likelihood of VH occurrence and the probability of requiring invasive treatment such as vitrectomy is higher in patients with VH occurrence.

In this study, 41.9% showed hyperpigmented spots after the induction therapy, which was almost similar to the previous study (47.1%)^19^. Also, larger SMHs tended to have hyperpigmented spots, which was consistent with the their study. Kim et al.^19^ described that hyperpigmented spots after the SMH can affect the course of the disease, which showed the low-incidence of re-activation and late re-activaiton. Because some patients in our study received proactive treatment, it was difficult to evaluate the time of recurrence. However, eyes with larger SMHs should be paid attention to the appearance of hyperpigmented spots and these eyes might need to be followed for long-term for the risk of reccurrence.

The limitations of the current study were its retrospective nature, the small sample size, and the non-comparative study design. Because the data for VH + group was based on only five eyes, it might be insufficient to say that the study has sufficient power for statistical analysis. Furthermore, the fact that different treatment methods were used between the IVA and IVBr groups after the initial loading injection was also a limitation of this study. Therefore, a large-scale randomized study is needed to confirm the optimal treatment for patients with SMHs. More invasive treatments such as displacement of SMHs with expansile gas or intravitreal tPA combined with gas showed favorable outcomes if patients were treated within 14 days^20,21^. Furthermore, vitrectomy seems to be associated with a higher rate of recurrent hemorrhages or retinal detachments as postoperative complications^22,23^. However, anti-VEGF treatments are minimally invasive and associated with a lower treatment burden. For example, patients who have difficulty maintaining prone positioning because of old age or hemiplegia or patients with a long symptom duration especially may be appropriate candidates for anti-VEGF therapies for SMHs related to AMD.

In conclusion, IVA and IVBr appeared to improve the functional outcome in patients with AMD with SMHs. A downside is that 8.1% of the patients developed a VH, which resulted in worse VA. IVA and IVBr might be useful to treat SMHs related to AMD. However, for cases with large SMH at baseline, it should be considered that VH may occur during the monotherapy treatment process using IVA or IVBr, and that achieving good visual outcomes may be difficult in some cases.

## Methods

We retrospectively studied 62 consecutive treatment-naïve eyes with SMHs that exceeded one DA, included the fovea secondary to AMD, and were treated with IVA or IVBr. All patients were treated initially at Yokohama City University Medical Center between April 2013 and May 2021. This institutional review board of the Yokohama City University Medical Center approved the study, which was conducted according to the tenets of the Declaration of Helsinki. All patients provided written informed consent before their medical record data were used in this research.

The inclusion criteria were the presence of a SMH exceeding one DA secondary to neovascular AMD determined by clinical findings and the availability of images obtained by spectral-domain optical coherence tomography (SD-OCT) (Spectralis Product Family Version 5.3, Heidelberg Engineering, Heidelberg, Germany), fluorescein angiography, and indocyanine green angiography (Spectralis Product Family Version 5.3 or California, Optos, Dunfermline, Scotland, UK). Patients were excluded who had been treated previously for AMD by laser photocoagulation, photodynamic therapy, vitrectomy, intravitreal injection of other anti-VEGF agents, or intravitreal steroids, and had a history of uncontrolled glaucoma, macular hole, diabetic retinopathy, uveitis, retinal vein occlusion, and rhegmatogenous retinal detachment.

All patients received three monthly intravitreal injections of IVA or IVBr during the loading phase. For patients treated with IVA, the injection was administered based on an as-needed or treat-and-extend regimen in the maintenance phase. Patients treated with IVBr received treatments every 12 weeks unless new fluid or a new hemorrhage developed. If that occurred, IVBr was administered every 8 weeks. If a VH developed after the treatment, injections were discontinued, and vitrectomy was performed.

We divided the patients into two groups depending on whether a VH had or had not developed (VH + group and VH − group, respectively) during the follow-up period. The main outcome measure was the changes in the best-corrected visual acuity (BCVA) in each group. Multiple regression analyses were performed to determine the correlations between the parameters, including age, sex, anti-VEGF (aflibercept or brolucizumab), development of VHs after treatment, use or no use of anticoagulants, AMD type (PCV or non-PCV), symptom duration, DAs, baseline BCVA, baseline CFT, baseline thickness of the SMH at the fovea, baseline thickness of the hemorrhagic PED at the fovea, and the differences between the pre- and post-injection BCVA 12 months post-treatment. The changes in the BCVA were used as the dependent variable. Furthermore, the factors that affected the development of VHs also were evaluated. The association between the development of VH and age, sex, type of anti-VEGF agents, use or no use of anticoagulants, AMD type, duration of symptoms, DAs, CFT, thickness of SMHs at the fovea, and the thickness of the hemorrhagic PEDs at the fovea as the risk factors that affected VH development also was investigated. Also, we evaluated the changes in the BCVA and the factors that affect the BCVA improvement at 12 months in patients with PCV as sub-analysis.

Furthermore, we evaluated the hyperpigmented spots appearing after the SMH using fundus photograph acquired 1–2 months after the third injection as Kim et al.^19^ described. The association between age, sex, type of anti-VEGF agents, development of VHs after treatment, use or no use of anticoagulants, AMD type, duration of symptoms, DAs, baseline BCVA, CFT, thickness of SMHs at the fovea, and the thickness of the hemorrhagic PEDs at the fovea as the factors that affected the hyperpigmented spots also was investigated.

The statistical analysis software used was Ekuseru-Toukei (Social Survey Research Information, Tokyo, Japan), and paired intergroup comparisons of BCVA were performed using the Wilcoxon signed-rank test. Factors affecting the BCVA changes were analyzed using multiple linear regression. Factors affecting VH development and hyperpigmented spots were analyzed using stepwise logistic analyses. *P* < 0.05 was considered significant.

## Data Availability

The datasets used during the current study are available from the corresponding author on reasonable request.
